# Supplementary material for: Chemical Modifications to Enhance the Drug Properties of a VIP Receptor Antagonist (ANT) Peptide
Source: Int J Mol Sci. 2024 Apr 16;25(8):4391. doi: 10.3390/ijms25084391 (PMC11050070; doi:10.3390/ijms25084391)
Supplement: Supplementary file 1 [file ijms-25-04391-s001.zip › ijms-2952253-supplementary.pdf]

Supplementary Materials:

1. HPLC and MS spectra 2

1.1. Peptide-PEG SDS-PAGE... 7

1.2. Additional Peptide Sequences.. 8

1.3. Plasma Stability T Cell Activation Study 9

1. HPLC and MS Spectra

Peptide	MW	6M^{6+} calc	6M^{6+} exp	5M^{5+} calc	5M^{5+} exp	4M^{4+} calc	4M^{4+} exp	3M^{3+} calc	3M^{3+} exp
**ANT308**	3466.16	578.70	578.67	694.23	694.20	867.55	867.50	1156.39	1156.33
**AcANT308**	3505.98	585.71	585.67	702.66	702.60	878.07	878.00	1170.42	1170.33
**ANT308K**	3620.27	604.39	604.35	725.06	725.02	906.07	906.02	1207.76	1207.70
**ANT308C13C17**	3425.86	571.98	572.15	686.18	686.58	857.47	857.72	1142.96	1143.30
**ANT308C13C17stp**	3530.23	589.38	589.33	707.05	706.99	883.56	883.49	1177.75	1177.65

ANT308

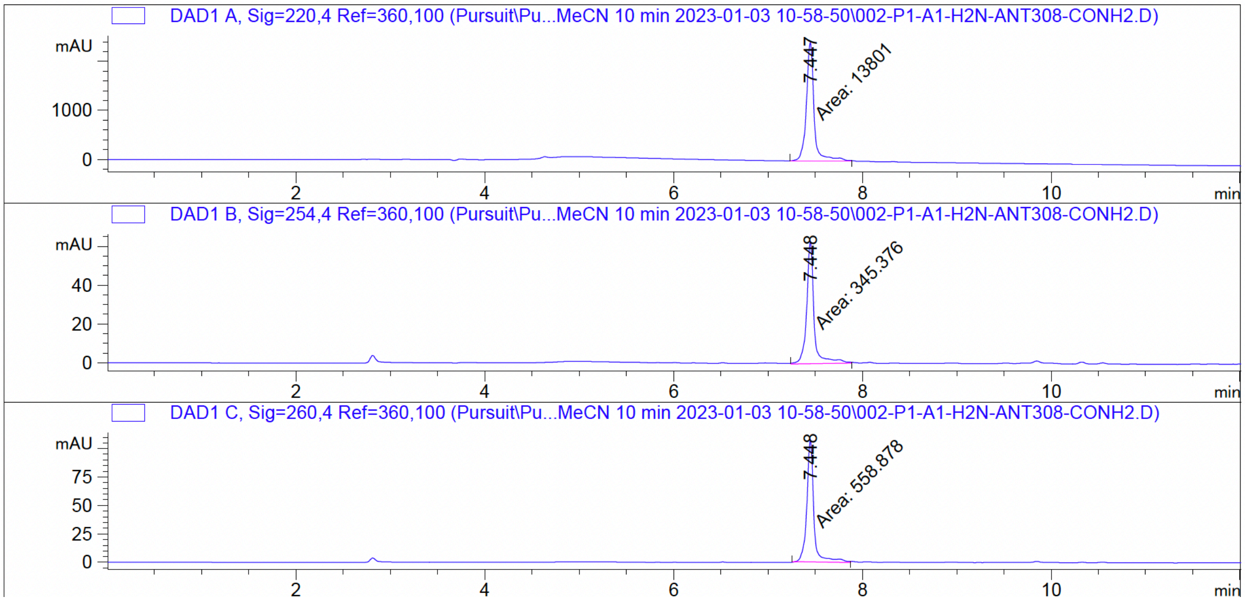

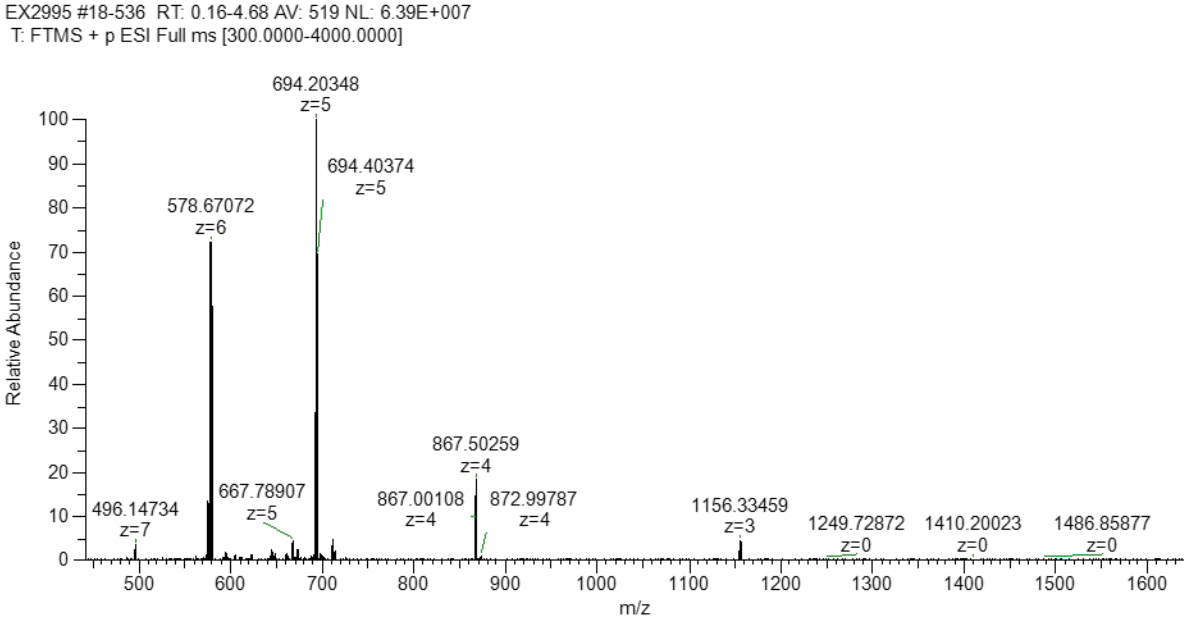
EX2995 #18-536 RT: 0.16-4.68 AV: 519 NL: 6.39E+007
T: FTMS + p ESI Full ms [300.0000-4000.0000]
Relative Abundance
m/z
694.20348 z=5
694.40374 z=5
578.67072 z=6
667.78907 z=5
496.14734 z=7
867.50259 z=4
867.00108 z=4
872.99787 z=4
1156.33459 z=3
1249.72872 z=0
1410.20023 z=0
1486.85877 z=0

Ac-ANT308

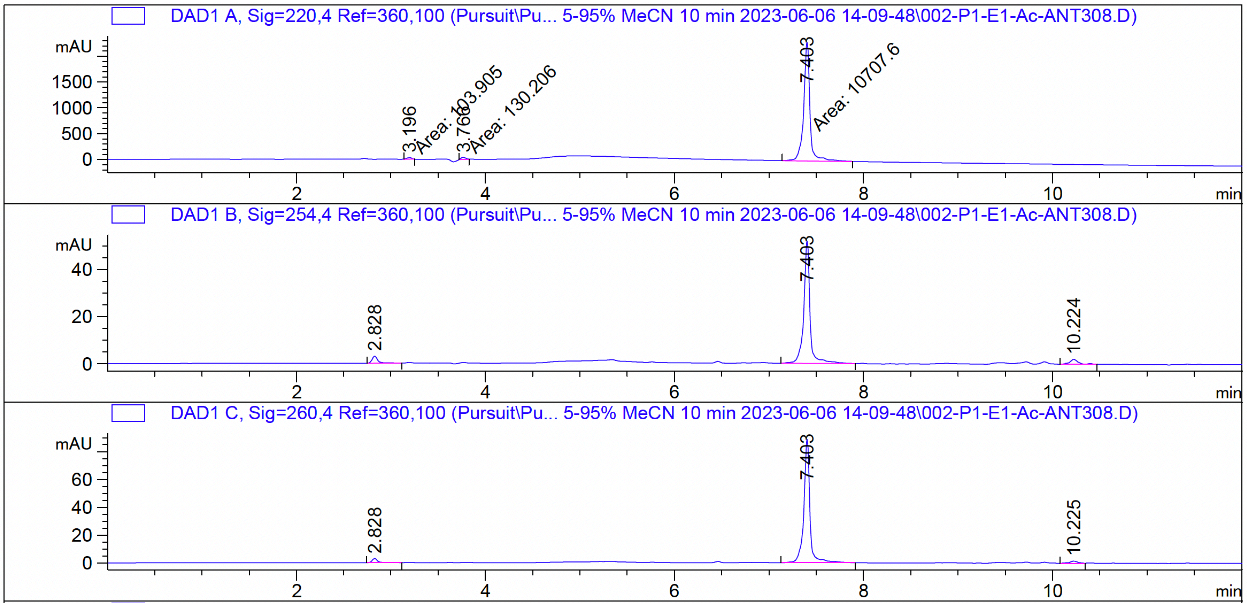
DAD1 A, Sig=220,4 Ref=360,100 (Pursuit\Pu... 5-95% MeCN 10 min 2023-06-06 14-09-48\002-P1-E1-Ac-ANT308.D)
mAU
3.196
Area: 103.905
3.768
Area: 130.206
7.403
Area: 10707.6
min
DAD1 B, Sig=254,4 Ref=360,100 (Pursuit\Pu... 5-95% MeCN 10 min 2023-06-06 14-09-48\002-P1-E1-Ac-ANT308.D)
mAU
2.828
7.403
10.224
min
DAD1 C, Sig=260,4 Ref=360,100 (Pursuit\Pu... 5-95% MeCN 10 min 2023-06-06 14-09-48\002-P1-E1-Ac-ANT308.D)
mAU
2.828
7.403
10.225
min

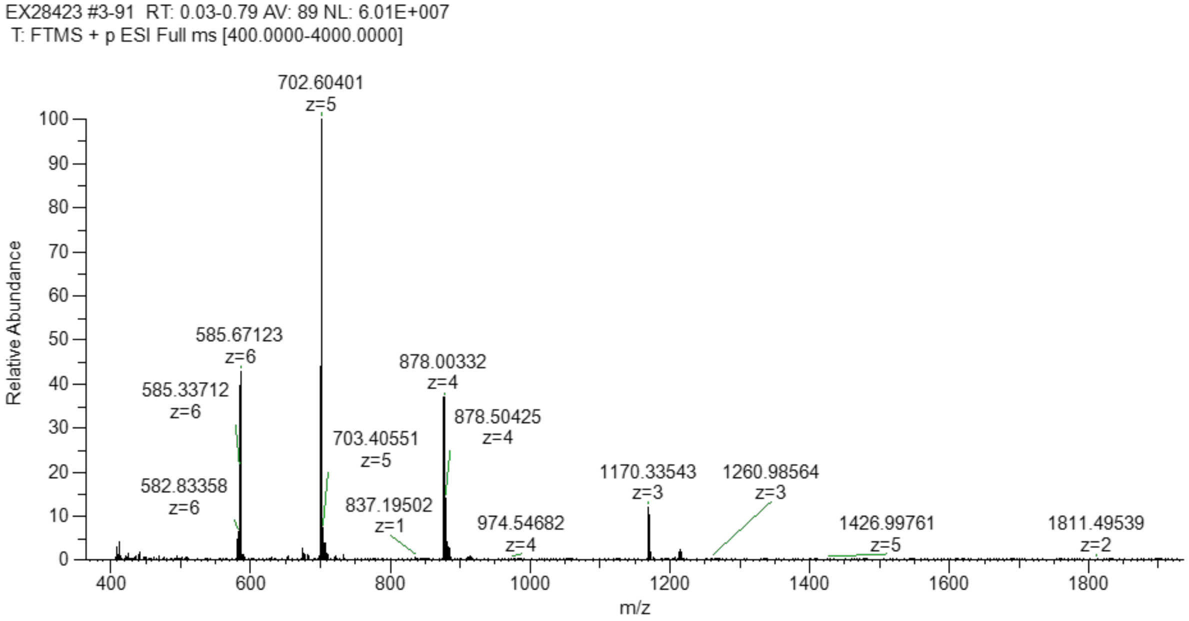
EX28423 #3-91 RT: 0.03-0.79 AV: 89 NL: 6.01E+007
T: FTMS + p ESI Full ms [400.0000-4000.0000]
Relative Abundance
m/z
702.60401 z=5
585.67123 z=6
585.33712 z=6
582.83358 z=6
878.00332 z=4
878.50425 z=4
703.40551 z=5
837.19502 z=1
974.54682 z=4
1170.33543 z=3
1260.98564 z=3
1426.99761 z=5
1811.49539 z=2

ANT308K$_{N3}$

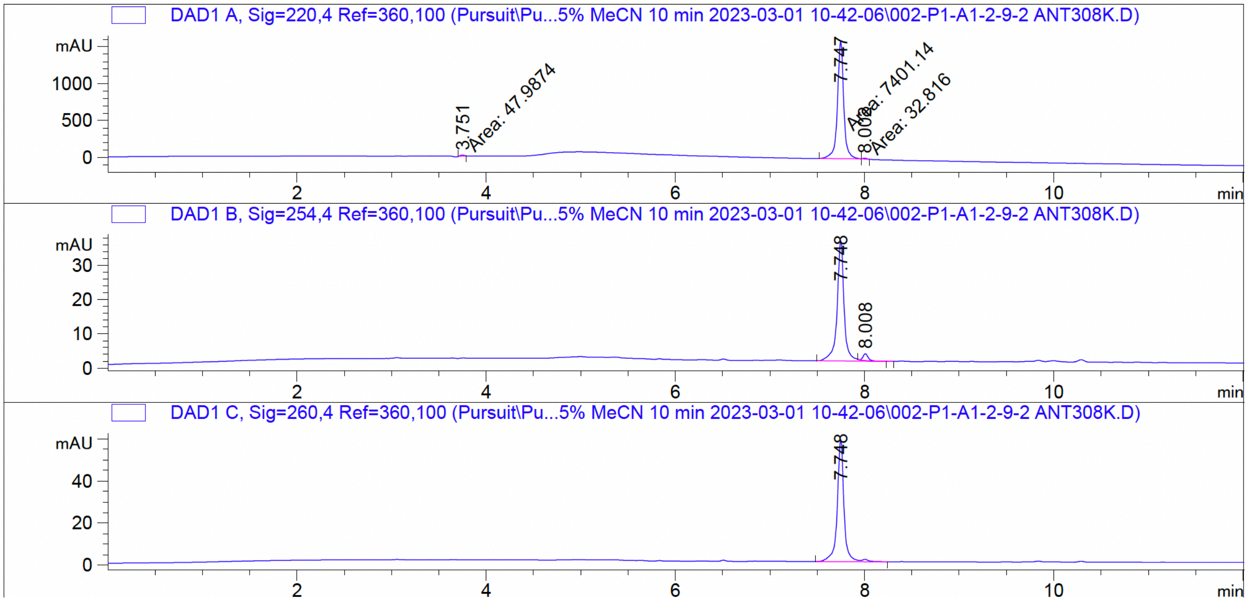
DAD1 A, Sig=220,4 Ref=360,100 (Pursuit\Pu...5% MeCN 10 min 2023-03-01 10-42-06\002-P1-A1-2-9-2 ANT308K.D)
mAU
3.751
Area: 47.9874
7.747
Area: 7401.14
8.008
Area: 32.816
min
DAD1 B, Sig=254,4 Ref=360,100 (Pursuit\Pu...5% MeCN 10 min 2023-03-01 10-42-06\002-P1-A1-2-9-2 ANT308K.D)
7.748
8.008
DAD1 C, Sig=260,4 Ref=360,100 (Pursuit\Pu...5% MeCN 10 min 2023-03-01 10-42-06\002-P1-A1-2-9-2 ANT308K.D)
7.748

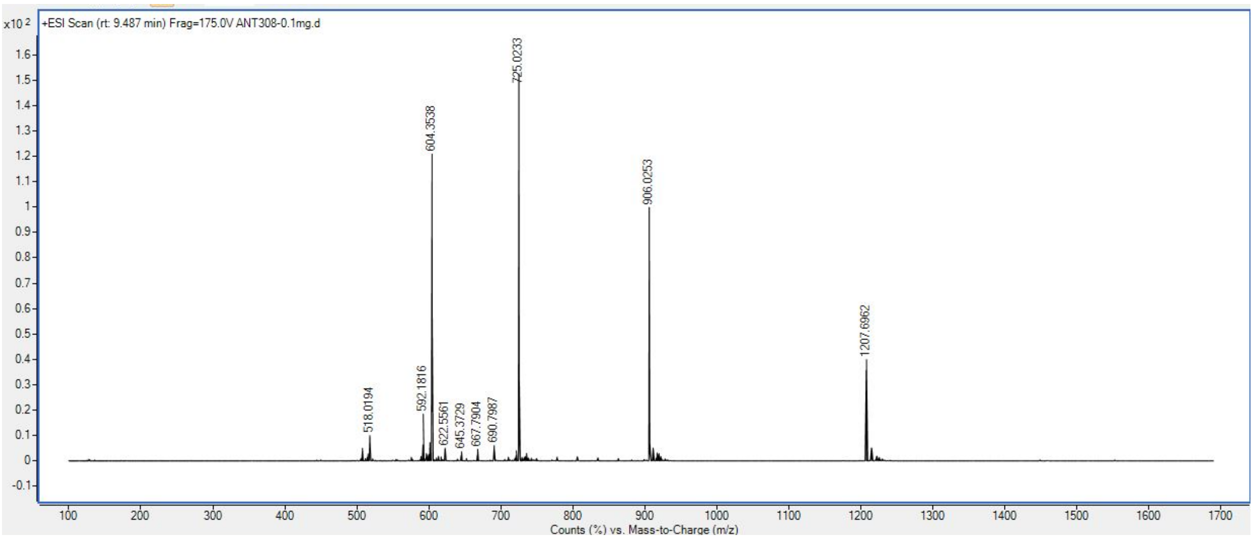

ANT308C13C17

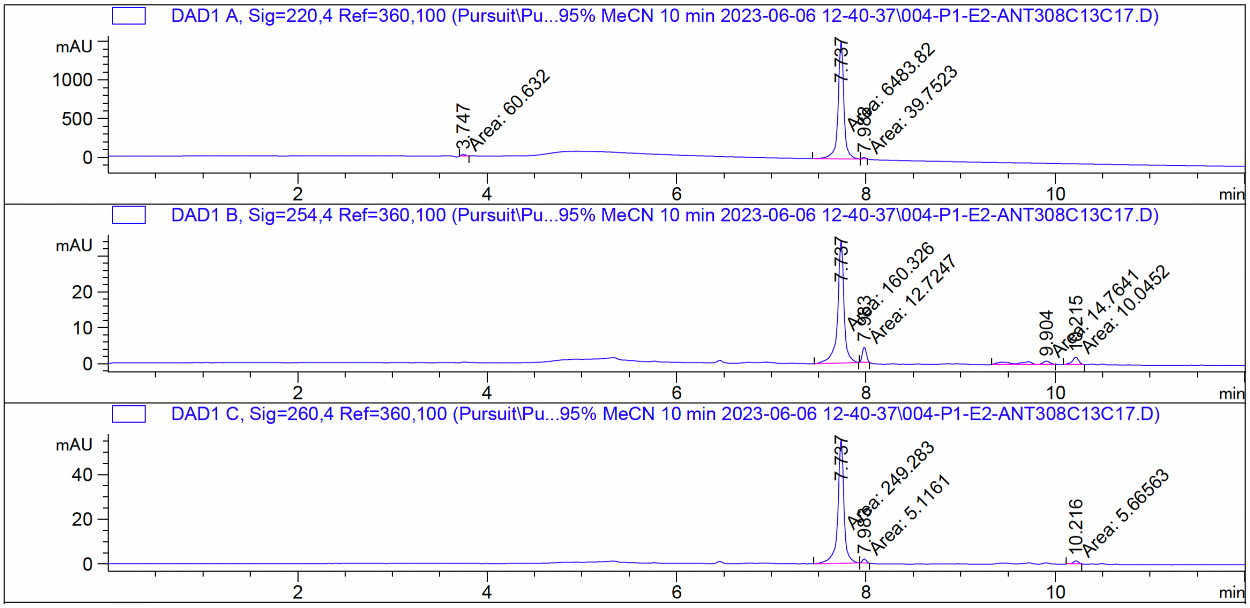

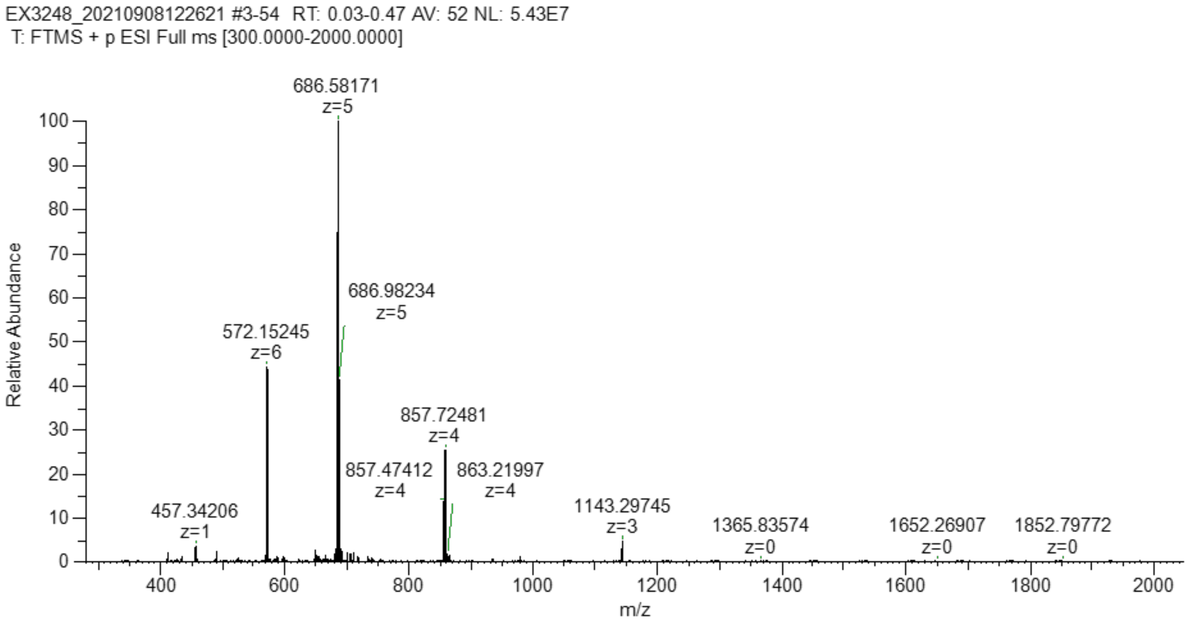

ANT308C13C17 stp

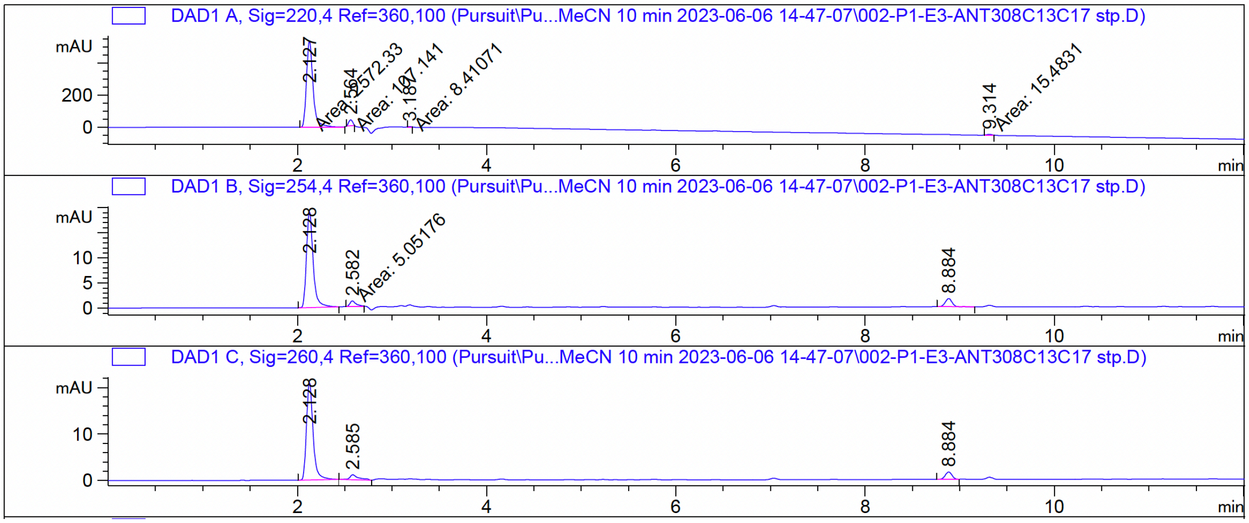

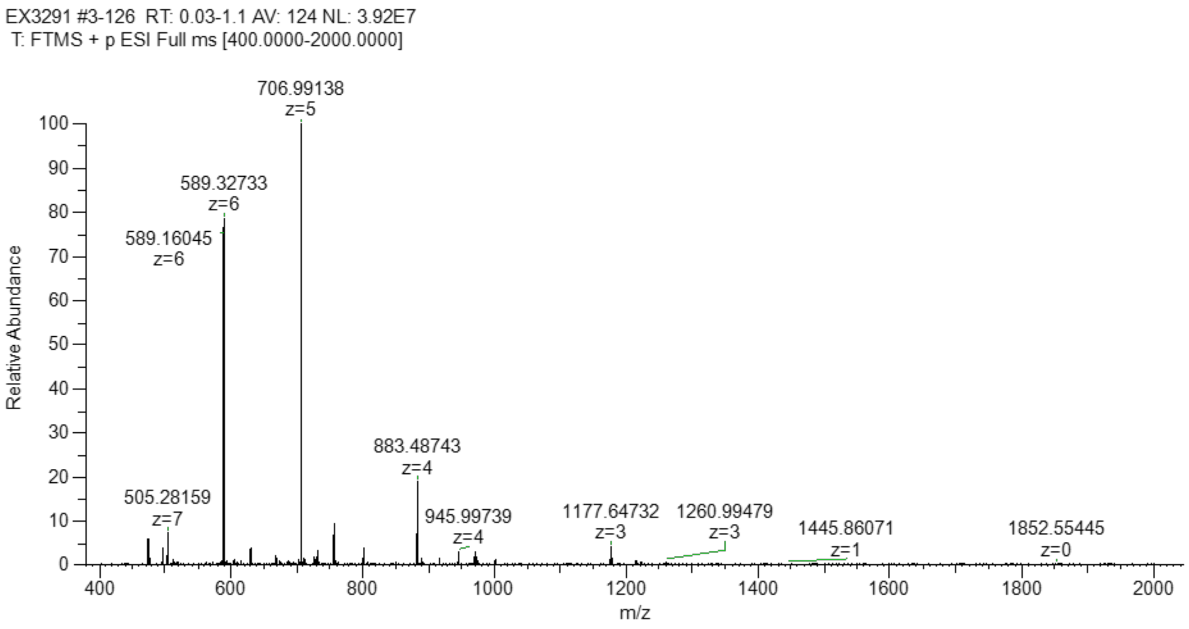

1.1 Peptide-PEG Analysis and Characterization

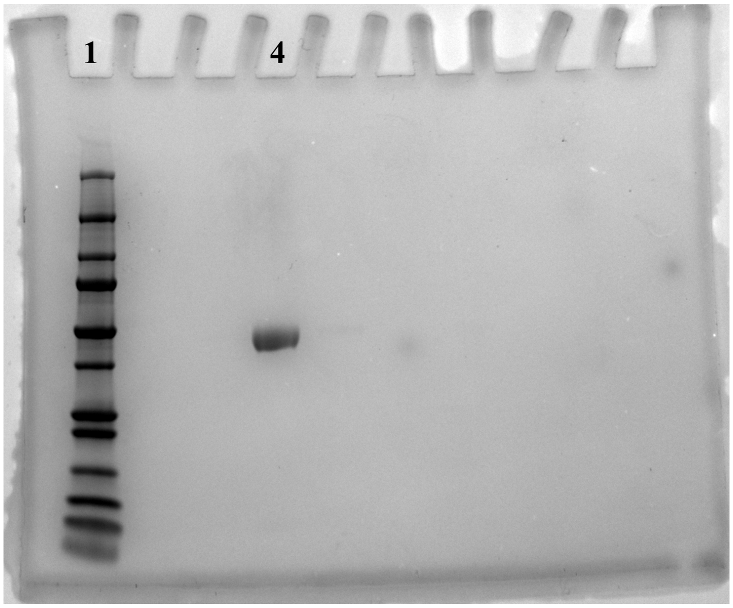

Lane	Sample
1	ladder
4	ANT308-PEG

1.2 Additional Peptide Sequences

Peptide controls used in the murine studies were purchased from RS Synthesis (Louisville, KY):

Fully SCRAM: YDDHTKLKINTARLVSAQNVFNMLKYR-$CONH_2$

1.3 Plasma Stability T Cell Activation Study

200 µM ANT308 or 200 µM ANT308K-PEG were mixed with healthy donors' plasma at 1:1 ratio, followed by incubation at 37ºC. Aliquots (150 µL) were collected at 24 hrs, 48 hrs, 72 hrs, and 96 hrs and stored at -80ºC before pooled human T cells activation assay. Assays were performed with three different donor's plasma.

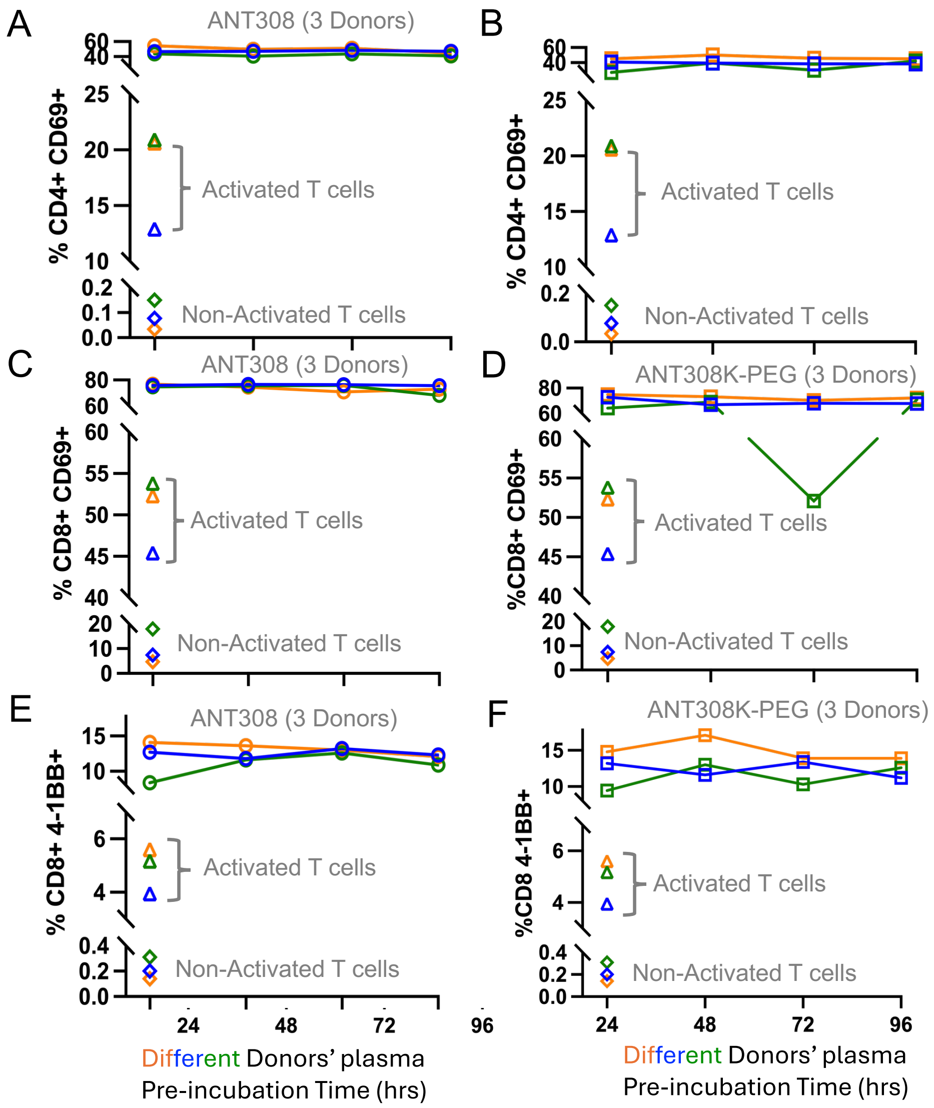

Figure S1A-F: Plasma stability and human T cells activation. After incubation with healthy donors' plasma at 37ºC for 24hrs, 48hrs, 72hrs, and 96hrs. 10 µM ANT308 (**A,C,E**) or 10 µM ANT308K-PEG (**B,D,F**) were directly mixed with isolated & pooled human T cells with the

presence of αCD3/CD28 activator and IL-2. 48hrs following activation, CD4 or CD9 T cell subsets were examined for CD69 and 4-1BB expressions.
